# Education of Idiots

**Published:** 1857-04-01

**Authors:** 


### EDUCATION OF IDIOTS.

On Wednesday, December 3, a meeting in support of the Asylum for Idiots, at Essex Hall, near Colchester, the object of which is to train and educate the idiot and imbecile, was holden in the Aldermen's Parlour, at the Town-hall, Cambridge. It was originally intended to hold the meeting in the large room, where specimens of the work of the inmates of the asylum, in the shape of fancy and useful articles, were laid out for inspection; but the state of the weather prevented many persons from being present, and the attendance was so small that the Aldermen's Parlour afforded ample accommodation.

The Rev. the Master of Jesus College was called upon to preside; he was supported by the Rev. E. Sidney, Rev. C. Clayton, R. M. Fawcett, Esq., R. Foster, Esq., Mr. Alderman Brimley, &c., and there were also present Professors Sedgwick and Selwyn, Archdeacon Harper, &c.

The Chairman having briefly stated the object of the meeting, the Rev. C. Clayton moved, and R. Foster, Esq. seconded, the first resolution, which was as follows:—

"That this meeting feels deep sympathy with the condition of the idiot and imbecile, and is strongly impressed with the value of the efforts now making for their amelioration and improvement, and with their claims upon the liberality of a Christian people."

The Rev. E. Sidney, Rector of Little Cornard, Suffolk, moved the second resolution:—

"That this meeting expresses its sincere gratification at the successful progress of the Asylum for Idiots, particularly with the branch so efficiently conducted at the Essex Hall, Colchester; heartily approves of the erection of the national model Asylum at Earlswood, near Reigate, and agrees to promote subscriptions to this interesting and needful charity."

He said it was three years that very day since he had the pleasure of submitting to a Cambridge audience some details concerning the Asylum for Idiots: since that time, the experiment had been carried on with more and more success. He stated at that time that it was but an experiment, and it was still so; but there were indications of great ultimate success. There was a time when the idiot was regarded as a poor mindless creature, to be fed and clothed, but kept out ot sight: his existence was almost ignored, he was the butt of the thoughtless, and his appearance was held to be revolting. But since the operation of such institutions as the one in question, the position of the idiot was changed; they had grappled with his infirmities, and made many conquests, and had prospect of many more. Science was casting her light upon the case of the idiot, and trying, in conjunction with Christian philanthropy, to find palliatives for his affliction. Now, what did they know about the idiot at the present time? One thing they had learnt—how to distinguish the idiot from the lunatic. Some were born idiots: in other cases they became so from some peculiar state of health. Some had their understanding undeveloped; these he should call idiots proper: others had their understanding partly developed, but were still very imbecile. With the first class, to render existence more comfortable was as much as could be done: with the second class, they could advance so far as to instil ideas of decency. With the mere simpleton they could do a great deal. The principle was to assume that the idiot had a mind like ourselves, but a feeble exhibition of mind due to an organization which is defective. A group of idiots would show every variety of disorder; it was impossible to mention any symptom of disorder which was not visible in a group of idiots. In the midst of these disorders there was some capacity, and the great thing was to observe what that capacity was. With respect to the capacities that idiots did not possess, they were mostly defective in speech, although some were voluble; some were defective in hearing, some in touch—to such an extent, indeed, that you might pinch them and they did not appear to suffer pain or to feel what you were doing. He named other physical defects, and spoke next of their mental defects, such as an absence of the powers of reflection, inference, and invention, and of all sense of morals, religion, and decency. In the midst of these defects, they had some power of distinguishing: if you put before an idiot at dinner-time a piece of board and a piece of bread, he could distinguish between them; he knew what was eatable from what was not. He had also some power of attention and conversation, and was capable of being influenced, for you always found an idiot yield to kindness and firmness. He had also some power of acquisition; and had, in short, feeble appetites, desires, affections, and dislikes.

As soon as a pupil entered the asylum, he was put under the care of one individual, who observed with great patience all that he did, and in an astonishingly short time became acquainted with his characteristics. The first thing was to correct the abnormal condition of health which idiots invariably laboured under, by careful physical training for the invigoration of the frame, particularly by such exercises as lead to the obedience of the body to the will. These things were the foundation of great improvement; for as the bodily health became invigorated, the mind developed itself. In a conversation which he (Mr. Sidney) had with Prince Albert, his Royal Highness said, "Don't you think fencing a good thing?" He (Mr. S.) replied that it was the best thing possible, because every well-directed muscular action implied a willing attention which improved the mind. They had attempted to teach the usual branches of education, and had succeeded; but it was necessary to proceed with extreme care. One boy was observed to like bowling; he could not read a single letter, but the master carved the letters on the top of the pins, and whenever the bowl knocked a pin down, he was not allowed to knock down another until he had named the letter, and thus he became able to read the Bible. How they taught reading, writing, and arithmetic was extraordinary; many curious appliances were used. One boy never could fix his eye upon anything, but he was brought to do so by catching a great glare, and children were now taught their letters by drawing them in phosphorus in a dark room. There was one boy who seemed to be quite unable to speak; but he was introduced three years ago into a room where there was a Christmas-tree, and immediately he cried out "tree, tree." But the best appliance for the instruction of idiots was the black board. The eye was the idiot's best power, and by the careful use of the black board, almost anything was taught by means of imitation lessons. The patience, care, and anxiety of the matter were almost inconceivable. Models were brought before them; they were taught with great difficulty to name objects. They were also taught mental arithmetic, which they learnt in a wonderful manner. The other day, he (Mr. Sidney) asked them this question—how much is one-half of two-thirds of three-farthings? After a few moments, a boy answered a farthing. The object lessons were exceedingly interesting. A piece of mahogany, for instance, was taken; they named it, and then you might engraft upon that almost anything. By these means much instruction was conveyed; but the patience of the teacher must never flag; the same thing must be repeated hundreds of times. The patience of the household at Essex Hall, and of Mr. Millard especially, was such as he had never witnessed before. It was an exceedingly difficult thing to select persons as teachers; and if idiot asylums should become common, there would be more trouble to get teachers than to get professors for this renowned university. They had taught the pupils good habits and proper demeanour, also something of morals; and the religious impression made upon their minds was wonderful indeed. They were also taught proper carriage, by the method of the drill; and so effectively, that two or three times he had told the officers of the camp near Colchester that he would match a company of idiots against a company of Rifles, or any other corps there. (Laughter). He was really not saying too much, for their drill was beautiful. The medical men were very kind; but the favourite doctor of all was the dentist, who won the heart of the idiot if he told him he must have a tooth out on Saturday.

With regard to statistics, there were 49 pupils at the institution at Earlswood, in Surrey; 26 were writers in books, and 23 on slates. There were 9 readers advanced, and 11 progressing; 10 were in the alphabet, and 10 drawing; 18 were doing imitation lessons; 6 were writers from imitation, and 9 were in arithmetic. The writers from dictation did it well; there was a peculiar aptitude at this in idiots sometimes; indeed, Goethe in his youth once dictated a poem upon Joseph and his Brethren to a poor imbecile. The principal trade was mat-making. At Essex Hall there are 20 boys in arithmetic. One of the officers in attendance upon Prince Albert, when he visited the asylum, asked a boy how much 1000 farthings made, which he answered readily. There were 33 in reading, 25 writers in books, and 13 writers on slates, and 12 in speaking lessons, which was a very curious part of the instruction. There was only one speechless idiot in the institution; many appeared to be speechless, but it was found that they were not so. One boy seemed to be unable to speak, but in the middle of the night he burst into one of the chants which he had heard during the day, and he could now speak well. Another boy, thought to be speechless, could write; one of his companions rubbed out what he had written, upon which he cried out, "Who has rubbed out my slate?" In singing lessons there were 13, and in drilling 44. The marching was beautiful; they marched to the sound of the bugle. There were 25 girls in the school.

In trades, there were gardeners and tailors, and some of the London tailors sent down garments to be made; you could never teach idiots to cut out a coat, but they could sew with great neatness; and if you were ever to go into the room amongst them, they would perhaps show great anxiety to sew on a button. He asked one boy how much he would charge to sew on a button, and he replied a shilling; but another boy said, "I'm ashamed of you; I would do it for the honour." They made mats; the demand for mats was so great that they could not be made fast enough; the Bishop of Rochester had all the mats for his palace at Danebury made at the asylum. The other day, a boy from Suffolk, who had been placed in the asylum by the Queen, sent a mat to Buckingham Palace, and it was graciously accepted. Alluding to some individual cases, he mentioned that of a boy who was remarkably sullen, and nearly speechless; he had now come out so wonderfully, that he had drawn two of the beautifully expressive pictures which might be seen in the next room; he was an admirable artist in wood, and was at this time earning wages as a carpenter, and was allowed pocket-money, because he earned more than was necessary for his support. He was a well-behaved proper person, of gentlemanly manners; still he was an idiot, and he (Mr. Sidney) did not believe that you could ever trust an idiot to take care of his own fortune; idiots had no knowledge of money, nor could it be imparted to them. When this boy was told he was to have 5*l.* a-year, nothing could persuade him that it was the same thing as if it were paid in monthly portions. The same boy made a beautiful model of a ship from a picture; also a black board, with grooves for the letters. He was likewise a most beautiful fencer; he (Mr. Sidney) once saw him fence with an officer who prided himself upon his skill, and he placed his foil upon the officer's heart. Another boy could be taught nothing at first; he was most surly, and so savage that when brought to the office, in the Poultry, London, he made such an uproar that a crowd collected. Visiting the asylum one day, in company with the wife of a baronet, some conversation arose about the making of slippers, and an inmate said he would make a pair for 5*s.* 6*d.*; this boy, however, said he would make a pair for the honour of working for such a lady. He was at first a very dangerous boy, and had three times tried to murder his own mother; now he was one of the most docile, gentle, and affectionate creatures ever seen. Several other instances of remarkable improvement were cited by the speaker. The last case showed that these poor creatures were capable of religious impressions. A girl was said to be self-willed and sullen, giving great trouble, and being resolved not to do anything she did not like. She became diligent and willing, showing great gratitude, and her mind opened to Scriptural truth in a wonderful way, seeming to verify the assurance that "blessed are the poor in spirit, for theirs is the kingdom of Heaven." She became ill and died; in her last hours she said, "I am going to Heaven, which is far better." As she was struggling in pain, she was heard to utter these words of the 23rd Psalm—"Thy rod and Thy staff they comfort me," and "I will arise and go to my Father." She died in hope, and left a wonderful example behind her.

Mr. Sidney proceeded to quote the testimony of the Bishop of Rochester, the Chaplain of Parkhurst Prison, and other gentlemen, to the excellence of the institution at Essex Hall. On the 19th of April in the present year,

Prince Albert visited it, and said what a pleasant place it was; the children sang "God save the Queen" upon the occasion. The Duke of Cambridge had also visited it, and was extremely pleased; and the Duke of Wellington spent three hours there, and sent a donation of 50*l*. The number of idiots was far greater than people supposed; it was calculated that there were 50,000 pauper idiots in the United kingdom. Was it not, then, a most interesting subject? They had educated the feeble germs of understanding; they had learnt these poor creatures to read, write, and cast accounts; they had made them love their benefactors, and led them to become acquainted with their God and Saviour. The task was a hard, but not a hopeless one. It was our duty not merely to foster talent, but to elicit capability of love and goodness, and show how great a reward, in a psychological point of view, flowed from educational and moral patience. With respect to what they had done, they had stopped deterioration in all, and shown improvement in many. Many had acquired self-control; most had acquired decency, and some had become useful and happy. There was a latent capacity that could be developed. He did hope, therefore, that Cambridge, whence had issued some of the brightest geniuses that the world ever saw, would not think it beneath her to assist the poor distressed idiot, but would believe that there is a mind to be expanded, and that it might be part of our probation here to lift up those feeble ones, to treat them as brethren, and to point out to them that hope which extends to brighter and better scenes. (Applause.)

R. M. Fawcett, Esq., seconded the resolution, and bore willing testimony to the able manner in which Mr. Sidney had handled the physiological and medical part of the question. He was happy also to bear testimony in some degree to the capability of idiots to acquire a certain amount of knowledge. Some years ago he was one of the visitors of the proposed lunatic asylum in this county, and he thought it his duty at that time to visit asylums in various parts of the country, during those little summer trips which he and others in Cambridge were in the habit of making. He was glad to observe great improvement in the treatment of lunatics, the system of cruel restraint being done away with; but he could not but regret seeing the old modes retained with idiots, who were allowed to sit in corners, or bask in the sun, poor, drivelling, useless cumberers of the institutions. At an asylum near York he observed a great improvement. It contained about 100 lunatics, all usefully employed, only three or four other domestics being required to do the work of the establishment. In a small court-yard, there were from twelve to twenty idiots working a windlass in full animal enjoyment, whereby they supplied the institution with water. It occurred to him then that if these poor creatures had been taken in hand when young, their mental faculties might have been in some measure developed. He was glad therefore to see such an institution as the present, intended for children and young persons who were idiots, and not for those adult pauper lunatics who were contemplated by the Act of Parliament under which county asylums were in process of construction. Mr. Fawcett concluded by drawing attention to some Scriptural texts upon the circular which the friends of the institution had distributed.

The Rev. Professor Sedgwick, who arrived late at the meeting, having been detained on his way from Norwich, proposed the next resolution:

"That the thanks of this meeting be given to the Rev. Edwin Sidney for his kind attendance and interesting statements relative to the Asylum for Idiots."

In the course of his few observations, he alluded to the case of a deplorable idiot whom he remembered as a boy in his native Yorkshire. That boy had no moral training whatever; and the ragamuffins of the place, by their jeers and annoyance, made him worse than could easily be conceived: yet he had extraordinary powers of calculation. The schoolmaster of the place would propose an extraordinary arithmetical problem to this brutal idiot, who would set to work, knit his brow, and bring out the correct numerical answer. How he did it he could tell no one. The Rev. Professor urged upon his hearers the duty of letting their charity flow in this new stream.

The Rev. Professor Selwyn seconded the motion, and warmly commended the institution to the consideration of the audience upon religious grounds. Alluding to Mr. Sidney's remark, that it was more difficult to find good teachers for idiots than good professors for the University of Cambridge, he noticed the importance to a teacher of a teachable audience: Niebuhr used to say to his class, "Ye are my wings." How difficult, then, must be the task of those who had to deal with these poor idiots!

Mr. Sidney having returned thanks, a vote of thanks to the Chairman and the Mayor was passed, on the motion of the Rev. F. Gell, seconded by the Rev. J. Y. Nicholson, which the Chairman acknowledged, and the meeting broke up.—*Cambridge Chronicle.*

